# Supplementary material for: Quantitative Multiplexed Proteomics Could Assist Therapeutic Decision Making in Non-Small Cell Lung Cancer Patients with Ambiguous ALK Test Results
Source: Cancers (Basel). 2021 May 12;13(10):2337. doi: 10.3390/cancers13102337 (PMC8150487; doi:10.3390/cancers13102337)
Supplement: Supplementary file 1 [file cancers-13-02337-s001.zip › cancers-1192797-supplementary.pdf]

Supplementary Materials: Quantitative Multiplexed Proteomics Could Assist Therapeutic Decision Making in Non-Small Cell Lung Cancer Patients with Ambiguous ALK Test Results

Ho Jung An, Eunkyung An, Shahrooz Rabizadeh, Wei-Li Liao, Jon Burrows, Todd Hembrough, Jin Hyung Kang, Chan Kwon Park and Tae-Jung Kim

Table S1. The results of selected reaction monitoring assay and evaluation of treatment responses.

Pt	FISH	ALK IHC		SRM (amol/ug)								Treatment	Best Response	PFS	Group
	ALK (%)	5A4	D5F3	ALK	MET	FRα	hENT1	RRM1	TUBB3	ERCC1	XRCC1		(%)	(m)	
1	B (19)	1+	P	245.8	332.1	820.0	142.2	269.2	2306.3	ND	424.0	Pemetrexed/cisplatin	SD (0)	11.1	4
2	P (55)	3+	P	289.3	ND	2266.2	ND	171.9	ND	ND	312.2	Pemetrexed/cisplatin	PR (−40)	10.3	4
3[1]	B (17)	0	N	ND	247.9	1494.8	121.2	119.5	ND	ND	370.4	Navelbine/cisplatin	N/A	12.4	4
4[1]	B (18)	0	N	ND	ND	689.7	ND	ND	ND	ND	377.4	Paclitaxel/carboplatin	N/A	19.5	4
5[2]	B (16)	0	N	ND	ND	467.7	ND	ND	2587.7	ND	408.9	Crizotinib	PD (+30)	0.9	0
6	P (52)	2+	P	577.5	ND	ND	ND	318.8	ND	ND	553.0	Pemetrexed/cisplatin	PD (+25)	1.8	2
7	B (16)	0	N	ND	ND	ND	ND	493.4	ND	204.6	717.0	Gemcitabine/carboplatin	PD (+25)	3.7	1
												Crizotinib	PD (+20)	1.5	0
8	B (16)	0	N	ND	615.8	933.8	ND	ND	3099.7	ND	472.2	Pemetrexed/cisplatin	PR (−45)	19.3	4
9	B (15)	0	N	ND	310.4	773.0	ND	331.3	ND	ND	480.2	Gemcitabine/carboplatin	PR (−56)	9.1	3
10	B (17)	0	N	ND	460.9	ND	ND	ND	2761.3	ND	670.3	Gemcitabine/cisplatin	SD (0)	21.8	3
11	P (44)	0	N	ND	594.0	1,512.7	ND	ND	1480.0	ND	396.4	Gemcitabine/carboplatin	PR (−39)	33.4	4
												Pemetrexed	SD (0)	30.3	4
												Crizotinib	PR (−30)	19.8	3
12[1]	P (38)	0	N[3]	162.6	ND	ND	ND	ND	4786.5	ND	56.02	Pemetrexed	PR (−80)	23.3	3
13[1]	B (17)	0	N	ND	ND	5636.7	ND	ND	668.5	ND	478.8	Gemcitabine/carboplatin	PR (−35)	2.0	4
14[2]	P (55)	0	N[3]	329.4	534.5	1399.5	ND	ND	3728.2	ND	480.7	Crizotinib	CR (−100)	13.8	4
15[1]	B (16)	0	N	ND	ND	1917.7	ND	ND	3543.5	ND	404.2	Navelbine/cisplatin	SD (0)	3.6	3
16[1]	B (17)	0	N	ND	ND	ND	ND	ND	4225.2	ND	480.1	No chemotherapy			
17[1]	B (16)	0	N	ND	ND	4926.2	ND	ND	3917.8	ND	812.0	No chemotherapy			
18[2]	B (18)	0	N	ND	ND	2963.7	257.2	ND	3780.8	ND	317.2	Crizotinib	PD (+30)	4.0	0
												Gemcitabine/carboplatin	PR (−37)	0.9	4
												LDK378	PD (+30)	5.0	0

19	P (24)	2+	P[4]	146.3	417.5	2735.7	ND	ND	6183.3	ND	409.3	Pemetrexed	SD (0)	43.6	4
												Crizotinib	PR (−56)	13.4	4
20[2]	P (32)	0	N	ND	ND	1887.3	351.0	ND	1728.5	ND	522.8	Crizotinib	SD (0)	3.7	0
21	P (46)	0	P	442.9	ND	ND	ND	502.2	9016.7	ND	648.7	Crizotinib	PR (−39)	3.2	3
												LDK378	PR (−44)	11.9	3
22	B (16)	0	N	ND	ND	1787.5	ND	ND	945.0	ND	527.5	Paclitaxel/carboplatin/ bevacizumab	PR (−45)	21.9	3

[1]Delayed fixation (overnight refrigeration), [2]Delayed fixation (>30 min, <overnight), [3]Case with weak negative staining, [4]Case with focal strong positive staining; Pt, patient; *ALK*, anaplastic lymphoma kinase; FISH, fluorescent in situ hybridization; IHC, immunohistochemistry; SRM, selected reaction monitoring; MET, hepatocyte growth factor receptor; FRα, folate receptor alpha; hENT1, human equilibrative nucleoside transporter 1; RRM1, ribonucleotide-diphosphate reductase M1; TUBB3, tubulin beta-3 chain; ERCC1, excision repair cross-complementation group 1; XRCC1, X-ray repair cross-complementing protein 1; PFS, progression-free survival; m, month; B, borderline positive; P, positive; N, negative; ND, not detected; SD, stable disease; PR, partial response; PD, progressive disease; CR, complete response; N/A, not available.
